# Mobile Technology Use and Its Association With Executive Functioning in Healthy Young Adults: A Systematic Review

**DOI:** 10.3389/fpsyg.2021.643542

**Published:** 2021-03-18

**Authors:** Rachel E. Warsaw, Andrew Jones, Abigail K. Rose, Alice Newton-Fenner, Sophie Alshukri, Suzanne H. Gage

**Affiliations:** Department of Psychology, Institute of Population Health Sciences, University of Liverpool, Liverpool, United Kingdom

**Keywords:** mobile technology, mobile devices, smartphones, executive function, cognition, brain

## Abstract

**Introduction:** Screen-based and mobile technology has grown at an unprecedented rate. However, little is understood about whether increased screen-use affects executive functioning (EF), the range of mental processes that aid goal attainment and facilitate the selection of appropriate behaviors. To examine this, a systematic review was conducted.

**Method:** This systematic review is reported in accordance with the Preferred Reporting Items for Systematic Reviews and Meta-Analyses (PRISMA) statement. A comprehensive literature search was conducted using Web of Science, MEDLINE, PsycINFO and Scopus databases to identify articles published between 2007 and March 2020, examining the use of mobile technologies on aspects of EF in healthy adults aged 18–35 years. In total 6079 articles were screened by title, and 39 screened by full text. Eight eligible papers were identified for inclusion. Our methods were pre-registered on the PROSPERO international prospective register of systematic reviews.

**Results:** A total of 438 participants were included across the eight studies. Five of the eight studies examined more than one EF. Five studies measured inhibition, and four studies measured decision-making. Smartphone use was negatively associated with inhibition and decision-making. Working memory performance was found to be improved by increased time engaging in video games and by refraining from smartphone use prior to bedtime. Quality assessments indicated high risk of methodological biases across the studies and a low quality of evidence for determining the relationship between technology use and executive functioning.

**Conclusions:** This review highlights the scarcity of the literature in this area. It presents a call for rigorous and objective research to further our understanding of the impact of mobile technology on different aspects of executive function.

Mobile devices have become integral to people's lives by offering a myriad of functions from communication, internet connectivity and the capacity to support additional applications (Heo et al., 2009; Lee and Calugar-Pop, 2019). Ownership of mobile devices has grown rapidly across the globe. For smartphones in particular, usage is high across developed countries. There is an estimated 851 million smartphone users in China, 345 million users in India, and 260 million users in the United States of America; the three largest markets of smartphone users as of September 2019 (O'Dea, 2020). Given their ubiquity, it is necessary to understand the potential impact of mobile devices on user's executive functioning.

To date, the research literature on the relationship between mobile device technology and executive functioning is equivocal. Some studies suggest a benefit of exposure, including improved task switching (Alzahabi and Becker, 2013) and attentional control (for review see: Green and Bavelier, 2012). Conversely, research has also demonstrated a negative relationship, including reduced task switching ability (Ophir et al., 2009); decreased attentional capacity (Ralph et al., 2014; Moisala et al., 2016) and working memory deficits (Sanbonmatsu et al., 2013; Cain et al., 2016; Uncapher et al., 2016). Moreover, smartphone use has been found to impair inhibition and working memory through inducing separation anxiety (Hartanto and Yang, 2016). Excessive smartphone use may also be related to reduced brain functional connectivity in regions associated with cognitive control: the orbitofrontal cortex (OFC), nucleus accumbens (NAcc) and midcingulate cortex (MCC) (Chun et al., 2018). Despite evidence of interesting and complex associations, there is a shortage of experimental longitudinal research in this area of the literature. This may be due to technological advancements in the industrial sector happening so quickly, that rigorous scientific and academic pursuits struggle to keep up.

A previous review (Wilmer et al., 2017) examined the existing literature with a focus on three facets of executive function: attention, memory and delay of gratification. Although the included evidence suggests a negative relationship between smartphone use and executive function, this may be unsubstantiated as methods were primarily correlational and made use of self-report data. Therefore, the literature lacks the longitudinal evidence needed to support claims of a detriment to memory or reward processing (Wilmer et al., 2017). With varying evidence across the literature, a clearer picture is needed on the association between mobile technology exposure and users' executive functions.

Executive functions (EF) are effortful mental processes which aid in the attainment of goals (Diamond, 2013). There are three core executive functions; inhibition and interference control, working memory, and cognitive flexibility (Miyake et al., 2000; Diamond, 2013). Inhibition and interference control contribute to regulation of one's behavior, attention, thoughts or emotions to respond to stimuli in an appropriate manner by overruling habitual or dominant responses. Working memory is the ability to hold information in the forefront of the mind after it is not perceptually present. Cognitive flexibility builds on the previous two EFs, enabling one to change their perspective, either spatially or interpersonally, allowing problems to be addressed in a new way or another's perspective to be understood. Taken together, these skills are essential for social (Mann et al., 2017) and academic success (Borella et al., 2010), and good health (Miller et al., 2011). Executive functions facilitate a range of goal-appropriate behaviors, including attending to important information (Psouni et al., 2019) and problem solving (Miller et al., 2020).

The National Institute for Health Research (NIHR) has published comprehensive guidelines on the effects of screen-based activities, with a focus on the impact on children and young people's mental health and well-being (Davies et al., 2019). Three components are highlighted for future research; the amount of time children spent in front of electronic screens for a variety of pursuits; the potential for exposure to harmful, inappropriate or illegal content; and companies' design architecture which encourages compulsive connectivity and engagement. The report concluded that the available scientific evidence is insufficient to draw conclusions and advise on optimal screen use.

Given the saturation of screen-based and mobile devices in society, objective scientific research into the effects of exposure on a variety of populations is needed to inform public policy. This review aimed to address the question of how exposure to mobile or video game technology affects executive functioning in healthy young adults. This was achieved by examining the existing peer-reviewed literature which investigated mobile device use and executive function in healthy adult samples, aged 18–35 years.

## Method

This systematic review is reported in accordance with the Preferred Reporting Items for Systematic Reviews and Meta-Analyses (PRISMA) statement (Moher et al., 2009 see Supplementary Material). An a priori protocol was published on the PROSPERO international prospective register of systematic reviews (CRD42019127003; https://www.crd.york.ac.uk/PROSPERO/).

### Eligibility Criteria

To qualify as eligible for inclusion, studies were required to investigate the use of mobile, or portable, technology, including but not limited to smartphones, video games and tablets. They had to include either between subject comparisons (e.g., groups with different extents of usage) or within participant comparisons (e.g., measures of any changes in usage between time points). The outcome measure of one or more aspect of executive functioning had to be independently assessed using validated self-reported or experimental methods outlined in the literature (e.g., Diamond, 2013). Participants had to be healthy adults, aged between 18 and 35 years old, recruited from the general population. This age group was chosen as they are less likely to be in cognitive decline (Salthouse, 2009; Murman, 2015), and this is in line with the NIHR's focus on children and young people. Any peer-reviewed published articles from between 2007 and March 2020 inclusive were considered. The start date was chosen as 2007 was the year Apple first introduced the iPhone. This marked a pivotal innovation as the features offered by the iPhone were more advanced than other devices and created the foundations of media consumption and mobile data use as we know it (Murphy, 2008).

### Information Sources and Search

The main search took place in April 2020, using four databases: Web of Science, MEDLINE, PsycINFO, and Scopus. Search terms were designed using scoping searches and adapted for suitability to each of the four databases; they included key words for different mobile technologies and aspects of executive function (see Table 1). The search strategy was piloted using Web of Science in March 2020. Scoping searches indicated that the inclusion of eligibility criteria, such as “healthy adults,” as a search term excluded some potentially relevant articles. Therefore, the search strategy was kept specific to technology and executive function terms, and the resulting literature was screened by hand for other eligibility criteria. RW performed the searches.

**Table 1:** Search strategy terms.

**#**	**Search strategy**
1	“mobile technolog*” OR smartphone OR “mobile phone*” OR “cell phone*” OR “screen time” OR touchscreen*
2	“executive function*” OR “executive control” OR cogniti* OR “self-regulation” OR “self-control” OR attention OR “working memory” OR “fluid intelligence” OR inhibit* OR impulsi* OR “impulse control” NOT biolog* OR “task switching” OR “problem solving” OR multitask* OR “delay of gratification” OR “delayed gratification” OR “delay discounting”
3	1 AND 2

### Study Selection

Three authors were responsible for the evaluation of articles for inclusion. RW screened titles and abstracts, with a random sample of 20% of the screenings cross-checked by AN-F and SA; there were no disagreements. Full texts of articles were screened by RW to identify those that met the eligibility criteria.

### Data Collection

Data was initially extracted by RW, and cross-checked by AN-F and SA. In instances where required data was not reported in the publication, corresponding authors were contacted to request this. Data extractions included country of origin, participants, mobile technology exposure (intervention), comparison, and executive function outcome (see Table 2).

**Table 2:** Summary of study characteristics.

**References**	**Title**	**Country**	**Participants**	**Mobile technology**	**Comparison**	**Executive function**
Chen et al. (2016)	General deficit in inhibitory control of excessive smartphone users: evidence from an event-related potential study	China	32. Excessive: *n* = 16 (7 males). Age: M = 19.50 ± 1.27. Normal: *n* = 16 (9 males). Age: M = 19.69 ± 1.30.	Smartphones	Between-subjects, excessive smartphone use group vs. Normal use group, categorized by SPAI scores	Inhibitory control
Donohue et al. (2012)	Cognitive pitfall! Videogame players are not immune to dual-task costs	USA	60. Video Game Players (VGP): *n* = 19 (no females). Non VGP: *n* = 26 (7 females). Overall age: M = 20.2, SD = 3.5. 52 males, 8 females.	Video games	Between-subjects, Video game players vs. non-video game players	Multi-tasking
Fortes et al. (2019)	Effect of exposure time to smartphone apps on passing decision-making in male soccer athletes	Brazil	20. All male. Age: M = 24.7 ± 3.6 years	Smartphones	Within-subjects, all participants took part in 4 conditions	Decision-making, inhibition
Fortes et al. (2020)	The effect of smartphones and playing video games on decision-making in soccer players: a crossover and randomized study	Brazil	25. All male. Age: M = 23.4 ± 2.8 years	Smartphones Video games	Within-subjects, all participants took part in 3 conditions	Decision-making, inhibition
Frost et al. (2019)	An examination of the potential lingering effects of smartphone use on cognition (study 2)	USA	50. M = 20.73. 14 males, 36 females.	Smartphones	Between-groups higher vs. lower smartphone use	Delayed gratification, problem solving
He et al. (2020)	Effect of restricting bedtime mobile phone use on sleep, arousal, mood, and working memory: a randomized pilot trial	China	38. Intervention group: *n* = 19. Age: M = 20.95 ± 2.07. 12 males, 7 females. Control group: *n* = 19. Age: M = 21.37 ± 2.63. 14 males, 5 females.	Smartphones	Between-subjects, intervention group vs. control group	Working memory
Huang et al. (2017)	The association between video game play and cognitive function: does gaming platform matter?	Canada	88. (50 females). VGP: *n* = 59. NVGP: *n* = 29. Age: M = 21.11, SD = 3.21.	Video games	Between-subjects, video game players, vs. non-video game players	Working memory, Inhibition
Tang et al. (2017)	Time is money: the decision making of smartphone high users in gain and loss intertemporal choice	China	125. (52 males). Age: M = 19.92, SD = 1.20.	Smartphones	Between-groups, low vs. medium vs. high smartphone users, categorized from SPAI scores	Decision-making, delay discounting, impulsivity

*SPAI, Smartphone Addiction Inventory*.

### Risk of Bias Assessment

The quality of the included papers was assessed using the Newcastle-Ottawa Scale (NOS) (Wells et al., 2000) adapted for cross-sectional studies. NOS was designed to evaluate the quality of non-randomized studies for inclusion in systematic reviews and meta-analyses. Studies were judged on three criteria; group selection, group comparability, and determination of the outcome of interest.

### Quality of Cumulative Evidence Assessment

The Grading of Recommendation, Assessment, Development and Evaluations (GRADE) framework was used to assess the quality of the body of evidence (Guyatt et al., 2011). Each study was assessed against downgrading and upgrading criteria within the domains of: factors which may decrease the quality of the evidence (e.g., methodological quality, directness of the evidence, heterogeneity, precision of reported results, and publication bias), and factors which may increase the quality (e.g., magnitude of effect, plausible confounds, and dose-response gradient). The results provided a rating of confidence in estimated effects within the studies, and therefore for the association between mobile technology and the measured aspect of executive function.

## Results

### Study Selection

**Figure 1:**
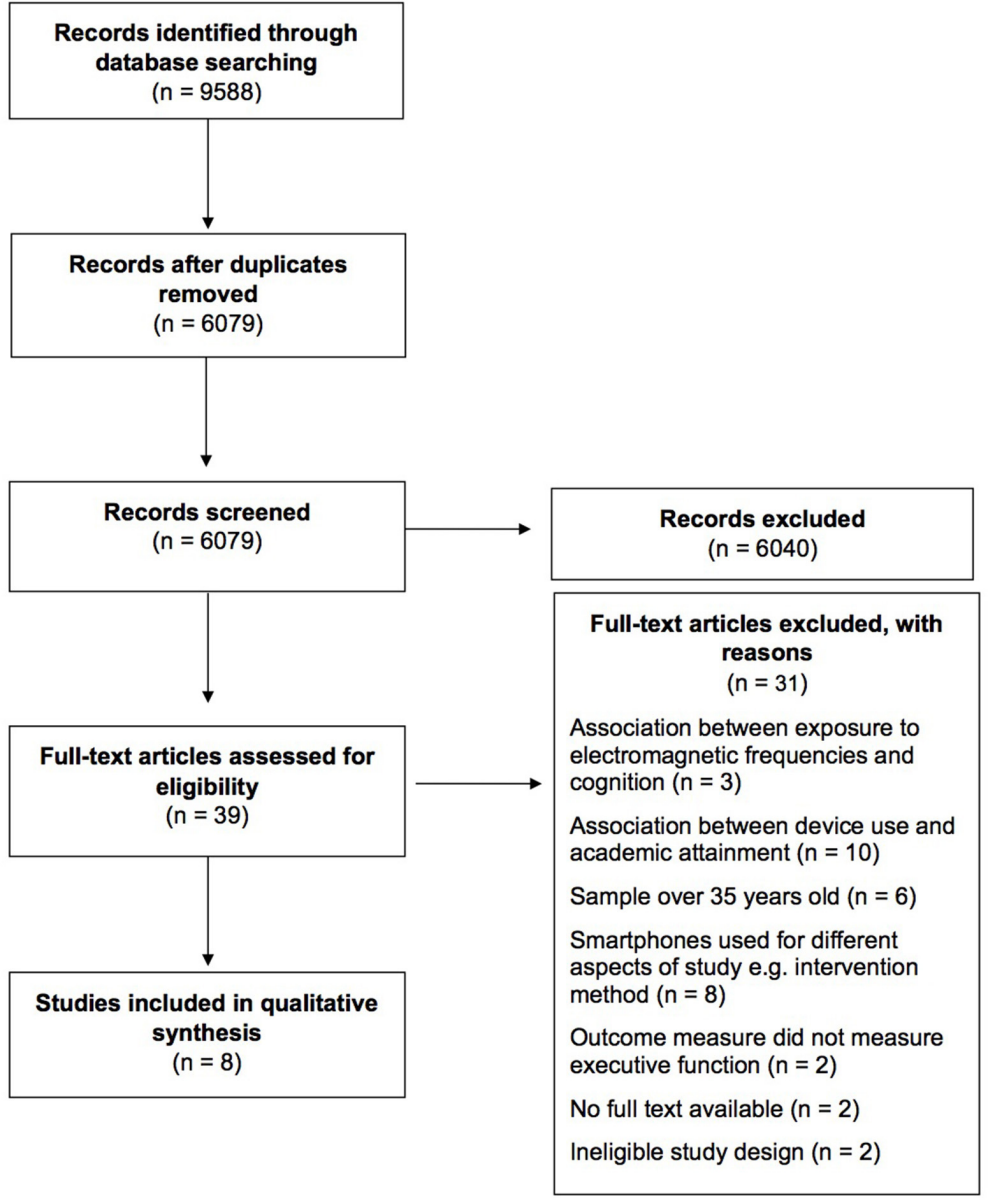
Flowchart of the selection of studies.

Once duplicates were removed, a total of 6,079 articles were identified from the searches. After screening, eight articles were identified as meeting the eligibility criteria (Donohue et al., 2012; Chen et al., 2016; Huang et al., 2017; Tang et al., 2017; Fortes et al., 2019, 2020; Frost et al., 2019; He et al., 2020). The study selection process is outlined in Figure 1.

### Study Characteristics

The number of participants in each study ranged from 20 (Fortes et al., 2019) to 125 individuals (Tang et al., 2017), with an average of 54. Participants were largely sampled from primarily student and university affiliated populations. All participants were aged between 18 and 35 years and had no reported history of any psychiatric or neurological disorders. Five articles assessed smartphones, two examined video games, and one article included both smartphones and video games (see Table 2).

### Risk of Bias in Included Studies

The cross-sectional adaptation of the Newcastle-Ottawa Scale (NOS) was used to screen for risk of methodological bias. Of the eight included studies, four were rated as “good,” and four as “satisfactory” based upon the three assessment areas: selection, comparability and outcome (see Table 3). According to the NOS, five of the included studies (Huang et al., 2017; Fortes et al., 2019, 2020; Frost et al., 2019; He et al., 2020) obtained comparable groups based on study design or analysis, by including valid control groups or reporting adjustments made for confounding variables. The remaining three articles (Donohue et al., 2012; Chen et al., 2016; Tang et al., 2017) did not report any adjustments to account for confounds.

**Table 3:** Summary of Newcastle-Ottawa Scale ratings and findings by article.

**References**	**Newcastle–Ottawa scale rating**	**Findings**
Chen et al. (2016)	Good	ERP N2 mean amplitude was larger for excessive smartphone users (upper 30% of SPAI scores), compared to controls (lower 30% of SPAI scores), *F*_(1, 30)_ = 11.67, *p* < 0.005, *η^2^p* = 0.28. demonstrates an electrophysiological inhibition deficit. No behavioral main or interaction effects of group were found.
Donohue et al. (2012)	Satisfactory	No interaction effects. VGP (played First Person Shooter [FPS] games in last 6 months, >average expertise) & NVGP (FPS games never played/not played in last 6 months, <average expertise) did not differ in multi-tasking/dual-task costs. VGP not protected from this through experience.
Fortes et al. (2019)	Good	There was a significant difference between smartphone exposure condition on Decision-Making Index (DMI) scores (*F =* 30.5, *p* < 0.001). DMI scores were significantly reduced at 30 min (*M* = 53.8, *SD* = 8.6) and 45 min (*M* = 51.4, *SD* = 10.1) compared to 15 min and control. An inhibition response time interaction was found (*F* = 21.4, *p* = 0.01). Inhibition was significantly impaired after 30 min (M = 6.2, SD = 1.4) and 45 min (M = 7.0, SD = 1.8) of smartphone use, compared to 15 mins and control.
Fortes et al. (2020)	Satisfactory	There was a significant difference between smartphone or video game exposure on decision-making performance (*F* = 23.6, *p* = 0.01, ES = 0.5). Both 30 min smartphone use (M = 57.2, SD = 9.1) and 30 min video game use (M = 60.7, SD = 9.6) had a detrimental effect on decision-making compared to control. There was a significant difference in inhibition response time (*F* = 32.5, *p* = 0.02), with 30 min smartphone use (M = 0.9, SD = 0.4) and 30 min video game use (M = 1.0, SD = 0.3) reducing inhibition performance compared to the control condition (M = 0.3, SD = 0.2).
Frost et al. (2019)	Satisfactory	Study 2: No difference between higher smartphone use group (≥ 5.5 h daily) and lower use group (≤2 h daily) was found for Delay of Gratification or problem solving.
He et al. (2020)	Satisfactory	Compared to the control group, intervention group demonstrated improved working memory performance after refraining from pre-bedtime smartphone use. Main effect of time in task accuracy found. Significant difference between intervention and control groups at post-test in the 1-back task (*F* = 5.02, *p* = 0.046) and 2-back task (*F* = 7.17, *p* = 0.036).
Huang et al. (2017)	Good	VGP (>5 h/week) had enhanced working memory compared to NVGP (<5 h/week), *F*_(9, 72)_ = 3,77, *p* = 0.001, *η^2^p* = 0.32. No effect on inhibition.
Tang et al. (2017)	Good	Participants categorized into high, medium and low groups using SPAI scores. Correlation between SPAI and BIS scores (*r* = 0.22, *p* = 0.01). High users showed irrational decision-making bias toward immediate rewards [*F*_(2, 122)_ = 6.76, *p* = 0.002, *η^2^p* = 0.100], and later penalties [*F*_(2, 122)_ = 3.335, *p* = 0.039, *η^2^p* = 0.052] compared to low users. High and medium users similar; suggesting a critical amount of smartphone usage to impact choices.

### GRADE Assessment

The body of evidence provided by the included articles for each outcome measure of executive function was GRADE assessed by one author (RW). Three of the included studies were rated “moderate,” three rated as “low,” and two rated as “very low” (see Table 4). Therefore, the evidence from these studies is likely to be weak regarding the associations between mobile technology and executive function.

**Table 4:** GRADE rating results for each executive function outcome.

**Outcome**	**Number of studies**	**GRADE**	
		**Rating**	**Reason**
Inhibition	5	Low	Imprecision of results. Exposure-response gradient.
Multi-tasking	1	Very Low	Imprecision of results. Design limitations.
Working memory	2	Moderate	Imprecision of results. Exposure-response gradient.
Decision-making	4	Low	Imprecision of results. Indirectness of evidence. Exposure-response gradient.
Problem solving	1	Low	Imprecision of results.

#### Inhibition

Five studies examined the association between mobile technology use and inhibition (Chen et al., 2016; Huang et al., 2017; Tang et al., 2017; Fortes et al., 2019, 2020). Chen et al. (2016) used behavioral and electrophysiological measures to assess inhibition in a total of 32 excessive and normal smartphone users (16 per group). Participants completed a novel Go-NoGo task, which had three cue contexts: blank, neutral and smartphone-related. Behavioral findings from the Go-NoGo revealed no group differences between excessive and normal users. However, there was an electrophysiological inhibition deficit between excessive and normal users (see Table 3).

Reduced inhibition was demonstrated by Tang et al. (2017), who used the Barratt Impulsiveness Scale (BIS) to assess impulsivity, and the Smartphone Addiction Inventory (SPAI) to divide 125 participants into high, medium and low usage groups. SPAI and BIS scores were positively correlated (*r* = 0.22, *p* = 0.01), suggesting increased smartphone use is associated with higher impulsivity. Further analysis demonstrated high smartphone users were more impulsive compared to low users, and that medium smartphone users were more impulsive compared to low users. No difference in impulsivity was found between high and medium use groups.

Fortes et al. (2019) assessed the effect of smartphone use on player's inhibition prior to a football match. Inhibition was quantified using the Stroop Task. Over 4 weeks, four conditions were completed: 0 (control), 15, 30, and 45 min of smartphone use exposure. Using a smartphone for 30 or 45 min was found to induce mental fatigue and impair inhibition, compared to the control and 15 min conditions.

The literature has also examined the association between video gaming and inhibition. Fortes et al. (2020) examined the impact of 30 min of video game use and smartphone use on inhibition performance, measured using the Stroop Task. Compared to the control of watching advertisement videos, 30 min of either video game play or smartphone use significantly increased response times to the Stroop Task, demonstrating reduced inhibition. In addition to this, Huang et al. (2017) used a Go-NoGo task to assess whether video game players (VGP) and non-video game players (NVGP) differed in inhibition. However, although VGPs demonstrated faster reaction times on average (~11 ms) there was no association between video game play and inhibition.

#### Decision Making

Tang et al. (2017) examined decision making in a sample of 125 participants. A delay discounting task assessed the decision-making process in high, medium and low groups of smartphone users, categorized by SPAI scores (see above). All participants completed both a gain and a loss task condition. In the gain condition, a choice had to be made between receiving smaller monetary rewards in the short term, or larger monetary rewards after a longer time delay. In the loss condition, a choice was made between whether to take the penalty sooner, or delay the loss. Participants in the high usage group and the medium usage group, respectively, had an increased preference for immediate rewards and postponed punishment, compared to the low usage group who chose delayed gratification in the gain condition, and to take penalties sooner in the loss condition.

Frost et al. (2019) used a self-report measure of delayed gratification to quantify the associations with smartphone use. In study two of their paper, participants were divided into lower (≤ 2 h) and higher (≥ 5.5 h) smartphone use groups and asked to ensure they met their assigned group's limit criteria. After 1 week of tracked smartphone use, they completed the Delayed Gratification Inventory (DGI-10), which scores across five domains: food, physical pleasure, social interaction, money and achievement. However, no difference of delayed gratification was found between lower and higher smartphone use groups.

In contrast, Fortes et al. (2019) investigated decision making during football matches. They used a within-subjects design to understand the effect of smartphone use exposure prior to a football match on passing decisions. Over 4 weeks, four conditions were completed: 0 (control), 15, 30, and 45 min of smartphone use exposure. Participants then completed a short Stroop task to measure their mental fatigue before playing a full football match. The game was video recorded and each pass was independently coded as appropriate or inappropriate by two researchers, who were blinded to the experimental conditions, to calculate a Decision-Making Index (DMI) score. At least 30 min of smartphone exposure was found to impair decision making; both 30 and 45 min exposure before game play reduced decision-making performance compared to 15 min and no exposure control.

Fortes et al. (2020) extended this research further to examine the effect of 30 min of exposure to smartphone or video game use on passing decisions in a football match. A third, control condition involved passively watching advertisement videos for 30 min. Twenty-five male football players took part in each of the three conditions, completing a short Stroop Task pre- and post- to the exposure condition, before playing a full football match. As before, the game was recorded and passing decisions independently coded to calculate a Decision-Making Index (DMI) score. Compared to the control condition, 30 min of exposure to either smartphones or video games was associated with significantly lower DMI scores, demonstrating impaired decision-making.

#### Problem Solving

Frost et al. (2019) investigated the relationship between smartphone use and problem solving. As previously, participants were divided into lower (≤ 2 h) and higher (≥ 5.5 h) smartphone use groups and asked to complete a self-report questionnaire on problem solving using the Modified Means End Problem Solving (MEPS). Independent judges rated participant's proposed solutions to hypothetical problems. However, there was no statistically significant group difference for problem solving (*d* = 0.08, *p* = 0.73).

#### Multi-Tasking

Donohue et al. (2012) examined the association between mobile technology use and multi-tasking, in a sample of 60 participants. The authors aimed to determine whether video game players (VGP) were better at multi-tasking than non-videogame players (NVGP). Participants were grouped based on their experience with First Person Shooter (FPS) games. All participants completed three tasks under single and dual-task conditions; computer simulated driving, multiple object tracking, and image search. The dual-task condition involved answering trivia questions while engaging in each task. There was no association between video gaming status and dual-task performance in any of the three tasks.

#### Working Memory

The association between video gaming and working memory (WM) performance was assessed by Huang et al. (2017), with a sample of 88 participants. Participants were grouped in to video game players (VGP) and non-video game players (NVGP) according to self-reported playing hours, and the Motivation for Video Game Use scale. WM was assessed using the N-Back task, which involved participants indicating whether a stimulus was presented in the same orientation as in a previous trial. The *N* refers to how many trials back they have to refer to, with difficulty increasing with each higher *N*. VGPs demonstrated increased task performance on the 1-Back and 2-Back trials, respectively, suggesting increased working memory performance compared to NVGPs.

The *N*-Back task was also used by He et al. (2020) to assess the impact of restricted mobile phone use prior to sleep. Participants completed baseline measurements before being divided into two groups. An intervention group had to refrain entirely from using their mobile phone for 30 min before their average bedtime. This was achieved either by parental control settings or, where these were unavailable on participant's phones, by instructions to turn their phone off 30 min prior to their bedtime. This was followed up by a researcher calling at any time to ensure compliance. A control group received no instructions regarding their phones. Post-test measures were then completed after 4 weeks. The intervention group demonstrated improved working memory performance in both the 1-Back and 2-Back trials compared to controls.

## Discussion

This systematic review aimed to assess the literature on mobile and video game technology exposure and the association with executive function in healthy adults aged 18–35. A total of eight papers examining five aspects of executive functioning were eligible for inclusion. Inhibition, decision-making, and working memory were outcome measures in more than one paper.

Increased smartphone use was found to be negatively associated with inhibition (Chen et al., 2016; Tang et al., 2017; Fortes et al., 2019, 2020) and decision-making (Tang et al., 2017; Fortes et al., 2019, 2020; Frost et al., 2019). According to Tang et al. (2017), there could be a critical threshold of device use, and use beyond this threshold contributes to impaired delayed gratification. In the present culture of information on demand, the instant access and connection enabled by smartphones is perhaps acclimatizing heavy smartphone users to expect immediate fulfillment of their commands, therefore reducing their capacity to delay gratification. Increased working memory performance was associated with refraining from smartphone use before sleep (He et al., 2020) and with playing video games (Huang et al., 2017). Video gaming was not associated with multitasking (Donohue et al., 2012). Contradicting evidence was found for video gaming and inhibition (Huang et al., 2017; Fortes et al., 2020).

A major issue of the identified evidence base is the poor quality of the studies. Three studies did not report any adjustments for confounding variables to ensure the comparability of groups (Donohue et al., 2012; Chen et al., 2016; Tang et al., 2017). Therefore, although the articles were rated as “good” or “satisfactory,” we are not able to determine the association between smartphones or video games and executive function from the evidence provided. The articles were also assessed using the GRADE criteria (Guyatt et al., 2011), which rated the body of evidence for each outcome as between “very low” and “moderate.” Articles primarily met downgrading criteria, such as: imprecise results reporting from an absence of confidence intervals (Donohue et al., 2012; Chen et al., 2016; Huang et al., 2017; Tang et al., 2017; Fortes et al., 2019, 2020; Frost et al., 2019; He et al., 2020); comparability issues, such as potentially homogenous groups (Donohue et al., 2012; Chen et al., 2016; Huang et al., 2017); and a reliance on self-report questionnaires (Tang et al., 2017; Frost et al., 2019).

A methodological issue in three of the eligible articles was homogeneity of groups (Donohue et al., 2012; Chen et al., 2016; Huang et al., 2017). Donohue et al. (2012) divided participants into two groups, Video Game Players and Non-Video Game Platers, based on their expertise in playing First Person Shooter (FPS) video games in the last 6 months. Using this arbitrary grouping, players of any other type of game may have been categorized as non-video game players, therefore diluting the comparison group. This may partly explain why there was no difference in dual-task costs between the two groups. Similarly, Huang et al. (2017) defined VGPs as playing for more than 5 h a week, and NVGP as playing < 5 h per week. Although they collected duration of game play per week, no mean duration game play for each group is reported. Therefore, the difference between these two groups could be minimal, which may invalidate the positive association between frequent video gaming and improved working memory. In general dichotomising groups as high vs. low exposure on a continuous variable is not recommended and can bring about misleading conclusions (Altman and Royston, 2006).

Chen et al. (2016) divided their participants into normal and excessive smartphone use groups using the self-report SPAI and a monitoring app installed on participant's smartphones. However, given the ubiquity of smartphones in daily life, “normal” and “excessive” use is hard to define. Although by all intentions the normal use participants were the comparison group, they still had access to and use of their smartphones. Therefore, the normal and excessive groups could have been too similar to examine the behavioral association between smartphone use and inhibition. Frost et al. (2019) also divided participants by extent of phone use, however their smartphone use groups were clearly distinct from one another (≤ 2 and ≥ 5.5 h) to examine the relationship with delayed gratification and problem solving.

A crucial methodological issue throughout is the reliance on self-report measures. Tang et al. (2017) quantified smartphone use and impulsivity using the SPAI and BIS, respectively, finding a negative association. However, self-reported and behavioral impulsivity have been demonstrated to be different to one another (Christiansen et al., 2012; Barnhart and Buelow, 2017). Furthermore, self-reported estimates may lead to underreporting of negatively perceived behaviors, e.g., video game play (Kahn et al., 2014), and in relation to executive functioning, correlate poorly with behavioral measures (Buchanan, 2016).

Frost et al. (2019) also quantified delay of gratification and problem solving using self-reported measures. The Delayed Gratification Inventory (DGI-10) is scored on a five-point Likert scale and covers five domains; food, physical pleasure, social interaction, money and achievement. Scores have been found to be associated with relevant behavior tendencies (Hoerger et al., 2011). Additionally, the Modified MEPS focuses on social problems, asking participants to provide solutions to social issues posed to them in small vignettes. It is possible that this limits the applications of these findings to only be applicable to social problem solving, rather than a wider variety of problem solving scenarios.

A strength of this review is that it is founded upon the existing literature and presents findings by the outcome measures they were intended to be contextualized with. The Miyake et al. (2000) framework of three core EFs was included at the beginning of this review (inhibition and interference control, working memory, and cognitive flexibility). It was a purposeful choice for the structure of the results to differ from this, to accurately reflect the complexity of the associations. However, this review is not without limitations. The included articles were of varying methodological quality, which should be kept in mind during interpretation. Additionally, there may be additional cognitive processes affected by mobile technology exposure, aside from executive functioning and, therefore, outside the remit of this paper.

Two of the five executive function outcomes reported here are only supported by one study (Donohue et al., 2012; Frost et al., 2019). The included studies are at risk for methodological bias. This collection of studies and inconclusive findings suggest that, at a point of increasing public concern about the associations between mobile technology and executive function, there was a gap in the literature to address. The urgency to fulfil this deficit perhaps resulted in studies of reduced quality. It is worthwhile to note that the quality of studies has improved over time, as the four more recent studies (Fortes et al., 2019, 2020; Frost et al., 2019; He et al., 2020) are conducted to a higher standard than the four earlier studies. They contain a pilot study for a randomized control trial (He et al., 2020), discrete groups of smartphone use (Frost et al., 2019) and repeated measures within-subject investigations (Fortes et al., 2019, 2020) to clarify the associations between smartphone and executive functions. This indicates that the literature in this area is improving in terms of methodological quality and therefore the reliability of estimates.

This systematic review highlights the inconclusive nature of the literature to date and acts as a call for rigorous and objective research on the association between mobile devices and executive functions.

## Data Availability Statement

The original contributions presented in the study are included in the article/Supplementary Material, further inquiries can be directed to the corresponding author/s.

## Author Contributions

RW conducted the main searches, article screening, quality assessments, and write-up. AJ, AR, and SG supervised and consulted in the design, preparation, and write up of the manuscript. AN-F and SA cross-checked article screenings by title and abstract. All authors contributed to the article and approved the submitted version.

## Conflict of Interest

The authors declare that the research was conducted in the absence of any commercial or financial relationships that could be construed as a potential conflict of interest.
